# Maternal Body Mass Index Associates with Prenatal Characteristics and Fecal Microbial Communities

**DOI:** 10.3390/nu16121881

**Published:** 2024-06-14

**Authors:** Nikita H. Nel, Eliot N. Haddad, Jean M. Kerver, Andrea E. Cassidy-Bushrow, Sarah S. Comstock

**Affiliations:** 1 Department of Food Science and Human Nutrition, Michigan State University, East Lansing, MI 48824, USA; 2 Department of Epidemiology and Biostatistics, Michigan State University, East Lansing, MI 48824, USA; 3 Department of Pediatrics and Human Development, College of Human Medicine, Michigan State University, East Lansing, MI 48824, USA; 4 Department of Public Health Sciences, Henry Ford Health, Detroit, MI 48202, USA

**Keywords:** BMI, gut microbiome, microbiota, pregnancy, prenatal health, bacteria

## Abstract

The maternal microbiome plays a vital role in shaping pregnancy outcomes, but there remains a substantial gap in understanding its precise relationships to maternal health, particularly in relation to potential effects of body mass index (BMI) on gut microbial diversity. The aim of this observational study was to assess maternal characteristics in association with pre-pregnancy BMI and to further assess microbial diversity in association with specific maternal characteristics. Eighty-four pregnant women were recruited during their third trimester of pregnancy from various prenatal clinics across the state of Michigan. The participants completed an enrollment questionnaire including self-reported pre-pregnancy BMI; stool samples were collected to assess the fecal microbial community composition. Pre-pregnancy obesity (BMI 30+) was associated (univariably) with antibiotic use before pregnancy, ever smoked, lower education level, and being unmarried. The gut microbiota alpha diversity was significantly different for pregnant women by pre-pregnancy BMI category (normal, overweight, obese). The beta diversity was unique for the gut microbiotas of pregnant women within each BMI category, by education level, and by marital status. Multivariable models revealed that pre-pregnancy BMI, maternal education, marital status, and maternal age were associated with the microbial diversity of the gut microbiota during pregnancy. These results give new insight into the relationship between a woman’s microbiome during pregnancy and their prenatal health, along with an understanding of the relationships between socioeconomic factors and microbial diversity.

## 1. Introduction

The maternal microbiome has become an increasingly important measure of maternal health and infant health outcomes. As the number of women with pre-pregnancy obesity has risen to almost a third (29%) of the United States’ population, it has become vital for healthcare providers to understand the mechanisms and effects pre-pregnancy obesity has on pregnancy and infant health [1]. The gut microbiota is responsible for digesting carbohydrates and other dietary components to produce vitamins, peptides, and short-chain fatty acids—factors essential for healthy fetal development [2,3]. The gut microbiome plays a critical role during pregnancy to help regulate a woman’s metabolism and immune system [4]. During birth, the infant gut microbiome undergoes bacterial colonization, which is shaped by the mother’s microbiome [5]. Thus, the direct vertical transfer of microbes from mother to infant has been identified as a potentially important factor in determining the health of the infant [6].

A growing body of evidence supports the hypothesis that a mother’s pre-pregnancy BMI and nutrition impacts the epigenetic profile of their offspring. Epigenetics characterizes the changes in gene expression and activity through DNA histone modifications such as methylation and acetylation [7]. During fetal development, maternal nutritional exposures play a vital role in establishing an infant’s epigenetic profile and future phenotypic changes, which impact their risk of disease [8]. The previous literature has identified numerous correlations between maternal pre-pregnancy BMI and methylation in newborn blood DNA [9]. While the mechanisms behind these associations are not well understood, some postulate that low levels of protein and fiber can alter one-carbon metabolism [10,11,12]. One-carbon metabolism involves various pathways that contribute to epigenetic expression such as the folate cycle and methionine cycle [13]. Through measuring changes in the gut microbiota, we may be able to further elucidate the complex interactions between maternal pre-pregnancy BMI and neonatal development.

Specifically, regarding prenatal BMI, there is contradictory evidence regarding its association with the gut microbiota during the latter third of pregnancy. Some studies have observed that gut bacterial diversity differs according to a person’s BMI status during this time in pregnancy [14,15,16]. For example, there is evidence that the gut microbiotas of women with obesity tend to have lower alpha diversity and higher levels of specific taxa like *Prevotella* [17]. Additionally, the ratio of gut *Firmicutes* to *Bacteroidetes* has been associated with BMI during pregnancy. Some studies have shown that the abundance of *Firmicutes* increases while the abundance of *Bacteroidetes* decreases in the gut with increasing BMI [18,19]. In contrast, other studies have observed that the pregnancy microbiota is similar regardless of BMI category [20]. While evidence suggests some connection between the pregnancy gut microbiome and BMI, more research is needed to understand (1) whether specific microbes have a direct association with BMI or other pre-pregnancy/pregnancy characteristics [14,16] as well as (2) the relationship between BMI and other pre-pregnancy/pregnancy characteristics.

The rise in the prevalence of obesity has been paralleled by an increase in neurodevelopmental and psychiatric disorders across the lifespan [21]. Many hypothesize that understanding microbial changes and detailing the nuanced microbial and molecular pathways in the gut–brain axis can reveal not only the underlying biological mechanisms behind these disorders but also potential targets for prevention and/or therapy [22,23]. The gut–brain axis is a bidirectional communication system that links the gastrointestinal tract and the central nervous system. This axis is regulated by neuronal, hormonal, and immunological signals [24]. Obesity is one of the many lifestyle factors that may mediate signaling within the gut–brain axis [23]. Children of women with obesity may have an increased risk of developing an intellectual disability or attention deficit hyperactivity disorder (ADHD) [25,26]. Research suggests that obesity increases the oxidative stress levels in the placenta while reducing blood flow, which can lead to restricted nutrient transfer to the developing fetus [27,28]. The detection of these important associations of obesity prior to and/or during pregnancy with child development have placed increased urgency on the need to understand the exposures that can alter the gut–brain axis and potentially prevent or treat such negative neurological health outcomes.

Using fecal samples and questionnaires collected from pregnant women during their third trimester of pregnancy, we aimed to address two main objectives. The first was to understand whether specific prenatal characteristics are associated with pre-pregnancy BMI. The second was to determine whether microbial diversity is associated with maternal pre-pregnancy BMI or other prenatal/pregnancy characteristics. Characterizing the pre-pregnancy/prenatal characteristics directly associated with the gut microbiome may help dietitians and clinicians identify risk factors, which could be targeted to ensure positive perinatal outcomes as well as positive health outcomes for infants.

## 2. Materials and Methods

### 2.1. Study Participants

The participants were recruited from the Michigan Archive for Research on Child Health (MARCH) prospective pregnancy cohort [29,30]. The MARCH study enrolled pregnant women from 21 prenatal clinics serving 11 birthing hospitals in the lower peninsula of the state of Michigan. The survey data and biological specimens were collected from pregnant women and their infants. The MARCH cohort is part of a nationwide collaboration, the Environmental Influences on Child Health Outcomes (ECHO) [31], and participating MARCH families are currently being followed for longitudinal data collection. The analysis described herein included a subset of pregnant women from the MARCH cohort who gave written informed consent for providing fecal samples and health survey/medical record data (n = 84). This study was approved by the human subjects’ research protection program at Michigan State University.

### 2.2. Data Collection

An enrollment questionnaire was administered to the pregnant women at their first prenatal study visit. The questionnaire captured each participant’s demographic information, pre-pregnancy health status, and health-related background information including medication usage, smoking status, etc.

Participants collected their own fecal sample using a fecal sample collection kit provided to them, for which the protocol has been previously described [14,32]. Briefly, collection kits were mailed to participants and included instructions for collecting a fecal sample, a Para Pak tube (Meridian Bioscience Inc., Cincinnati, OH, USA) for collection, and return postage for participants to return the sample. The samples were mailed, and upon sample arrival at the lab, they were aliquotted and stored at −80 °C.

### 2.3. Maternal Pre-Pregnancy Body Mass Index Classification

The participants pre-pregnancy BMI (kg/m^2^) was calculated using self-reported height and weight values. The participants were then classified into the BMI categories, normal (17 ≤ BMI < 25), overweight (25 ≤ BMI < 30), and obese (BMI ≥ 30) based on the Centers for Disease Control and Prevention BMI categories [33]. All of the participants had a BMI greater than 17, so the BMI category of underweight was excluded from our analysis.

### 2.4. DNA Extraction and Amplification

The protocol as described in Sugino et al., 2019 [14] was followed for DNA extraction and amplification of the V4 region of the 16S rRNA gene and sequencing. Following the mothur wet lab documentation, barcoded primers were used to amplify the V4 region of the 16S rRNA gene by PCR [34]. The successfully amplified triplicates were pooled and purified using Agencourt AMPure XP beads (Beckman Coulter, Brea, CA, USA). The Michigan State University Research Technology Support Facility Genomics Core sequenced the 16S rRNA libraries using paired-end, 250 base-pair sequencing on an Illumina MiSeq instrument using V2 chemistry (Illumina, San Diego, CA, USA).

The sequence reads were processed in mothur, on the high-performance computing cluster at Michigan State University, using the Illumina MiSeq SOP [34]. The sample reads were rarefied to 15,000 sequencing reads per sample and adequate community coverage was confirmed using rarefaction curves. Operational taxonomic unit (OTU) taxonomies were assigned by phylotype in mothur using the SILVA reference taxonomy (release V132) [35].

### 2.5. Statistical Analysis

The participant’s pre-pregnancy characteristics were compared between BMI groups using a Pearson’s chi-squared test of independence. Specific differences between each BMI group were identified using a pairwise test of independence for nominal data. Gut microbial diversity (alpha and beta) indices were calculated using the vegan package [36] in R version 4.3.0 [37].

The alpha diversity measures the bacterial diversity within a single community or organism. Herein, the alpha diversity is reported for three alpha diversity indices, Chao1, Shannon, and inverse Simpson. A Shapiro–Wilk test was performed to determine the normality of the data. Then alpha diversity differences between groups were tested. A Wilcoxon rank test was used to assess differences in the alpha diversity by antibiotic exposure, medication usage, mom age group, smoking status, college graduation status, allergen diagnosis, marital status, and car/home ownership. A Kruskal–Wallis test was used to test differences in the alpha diversity by pre-pregnancy BMI status and racial demographic groups. Post hoc comparisons for the Chao1 and Shannon indices were performed using a Dunn’s test for multiple comparisons. An ANOVA or *t*-test was performed for inverse Simpson index comparisons between groups. Tukey’s honest significant difference test was used for a post hoc comparison for the inverse Simpson index between pre-pregnancy BMI categories.

The beta diversity measures the bacterial diversity across two or more groups. Two beta diversity metrics were calculated: Sorensen and Bray–Curtis. The Beta diversity was visualized using a principal coordinate analysis plot (PCoA) in R [37] using the vegan package [36]. Permutational multivariate analysis of variance (PERMANOVA) and permutation of dispersion (PERMDISP) were performed for both Sorensen and Bray–Curtis to test for differences in the beta diversity. A negative binomial model in the MASS package [38] was used to compare the relative abundance of individual taxa with a >1% relative average abundance between BMI categories as well as by other prenatal characteristics.

In multivariable analysis, selected prenatal characteristics were added to a multiple linear model (function lm and ANOVA in R version 4.2.0). The alpha diversity models were analyzed using a type II ANOVA. The beta diversity models were analyzed using PERMANOVA (adonis2 function in the vegan package). The results were considered significant when the *p*-value was at or below the alpha level of 0.05.

## 3. Results

### 3.1. Participant Characteristics

We collected questionnaire responses from 84 women and compared pre-pregnancy characteristics by the BMI category of the participants (Table 1). The majority of participants n = 74 (88.1%) identified as white and n = 58 (69.9%) had a bachelor’s degree or higher. According to pre-pregnancy BMI, there were 44 women (52.3%) with a normal BMI, 17 women (20.2%) with an overweight BMI, and 23 (27.4%) women with an obese BMI. The maternal age was recorded for 81 participants and was similar across BMI categories with an overall average age of 31.5 years. The maternal age ranged from 20 to 52 years, with 41 participants having a maternal age ≤ 31 years and 40 with a maternal age >31 years. Thus, 31 years was set as a cutoff. Women with either overweight or obesity pre-pregnancy were significantly more likely to have used antibiotics before their pregnancy (*p* = 0.001). Women with obesity were significantly more likely to have ever smoked cigarettes (*p* = 0.006) and to be unmarried (*p* = 0.004) and were significantly less likely to be a college graduate (*p* = 0.024) or own stocks or bonds (*p* = 0.048).

### 3.2. Alpha Diversity

The third-trimester fecal alpha diversity significantly differed by pre-pregnancy BMI, maternal age, education level, marital status, and ownership of bonds status. Women with obesity prior to becoming pregnant had fecal microbial communities that were significantly more even (inverse Simpson) and had a lower diversity (Shannon) than those who had a normal or overweight BMI (Table 2). The participants had significantly less even (inverse Simpson) and more diverse (Shannon) fecal microbial communities at the genus level if they were over 31 years of age, a college graduate, married, and owned bonds (Table 2). The richness (Chao1) of the maternal fecal microbial community was similar regardless of the prenatal characteristic considered (Table 2). The other Table 1 characteristics were not associated with any alpha diversity metrics of the pregnancy gut microbiota.

### 3.3. Beta Diversity

**Figure 1:**
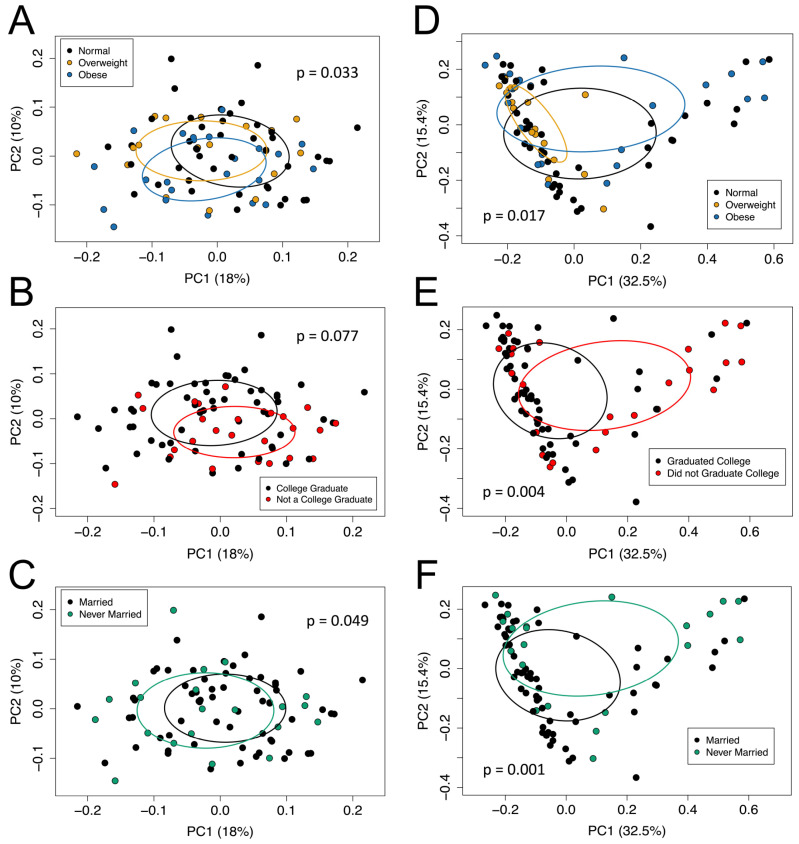
The beta diversity of the fecal microbiota samples was measured using the (**A**–**C**) Sorensen index and (**D**–**F**) Bray–Curtis index according to participants’ pre-pregnancy BMI category (**A**,**D**), education level (**B**,**E**), and marital status (**C**,**F**). The percent variation explained by each axis is in parenthesis, calculated from the PCoA eigen values. Ellipses are around the mean location of each maternal pre-pregnancy BMI category (**A**,**D**), education level status (**B**,**E**), and marital status (**C**,**F**). Each dot represents the composition of the gastrointestinal microbiota of one participant. Panel (**A**): PERMANOVA, *p* = 0.033; PERMDISP, *p* = 0.166. Panel (**B**): PERMANOVA, *p* = 0.077; PERMDISP, *p* = 0.889. Panel (**C**): PERMANOVA, *p* = 0.049; PERMDISP, *p* = 0.893. Panel (**D**): PERMANOVA, *p* = 0.017; PERMDISP, *p* = 0.015. Panel (**E**): PERMANOVA, *p* = 0.004; PERMDISP, *p* = 0.016. Panel (**F**): PERMANOVA, *p* = 0.001; PERMDISP, *p* = 0.015.

The pregnancy microbial community composition (Sorensen) was significantly different at the genus level when the participant’s fecal microbial communities were categorized by pre-pregnancy BMI (Figure 1A), education level (Figure 1B), or marital status (Figure 1C). Similarly, the pregnancy microbial community structure (Bray–Curtis) was significantly different at the genus level when the participant’s fecal microbial communities were categorized by pre-pregnancy BMI (Figure 1D), education level (Figure 1E), or marital status (Figure 1F). Additionally, the PERMDISP was significant for the Bray–Curtis dissimilarity matrix according to pre-pregnancy BMI (*p* < 0.05). The PERMDISP was significant (*p* < 0.05) in the Sorensen index and Bray–Curtis index for both education level and marital status.

### 3.4. Differences in Bacterial Taxa

**Figure 2:**
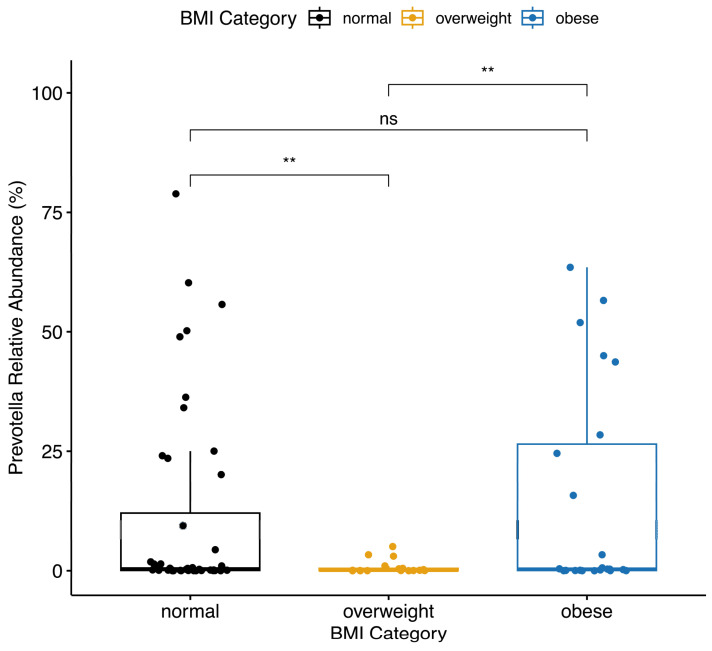
Women with a BMI category of overweight prior to becoming pregnant had significantly lower *Prevotella* relative abundance than women with normal weight or obesity prior to becoming pregnant. Boxplot center lines indicate median; boxes indicate interquartile ranges; and whiskers indicate 1.5× the interquartile ranges. ** = *p* < 0.01, ns = *p* > 0.05.

The maternal pre-pregnancy BMI category was associated with the relative abundance of *Prevotella*. Women who were overweight prior to their pregnancy had a significantly lower (*p* < 0.01) mean relative abundance of *Prevotella* (mean ± SD, 0.8% ± 1.5%) compared to women with normal weight (mean ± SD, 11% ± 20.1%) or women with obesity (mean ± SD, 14.6% ± 22%) (Figure 2). There was no significant difference in the *Firmicutes*-to-*Bacteroidetes* ratio by pre-pregnancy BMI category or any other pre-pregnancy characteristics tested. Smoking status and asthma diagnosis were related to the relative abundance of *Blautia*. Those who ever smoked had a significantly higher (*p* < 0.01) mean relative abundance of *Blautia* (mean ± SD, 2.9% ± 4.3%) compared to non-smokers (mean ± SD, 1.2% ± 1%). Similarly, those with asthma had significantly higher (*p* < 0.01) levels of *Blautia* (mean ± SD, 2.3% ± 3.5%) than those who did not have asthma (mean ± SD, 1.2% ± 1%). Marital status was significantly associated with *Lachnospiraceae.* Those who were married had a significantly higher (*p* < 0.05) relative abundance of *Lachnospiraceae* (mean ± SD, 8.2% ± 4%) compared to those who were unmarried (mean ± SD, 5.2% ± 3.5%).

### 3.5. Multivariable Analysis

A multivariable analysis was performed to determine which prenatal characteristics were associated with microbial diversity. Our multivariable model accounted for pre-pregnancy BMI category, antibiotic use, smoking status, marital status, college graduation status, and maternal age.

#### 3.5.1. Alpha Diversity

We assessed whether there was a difference in the results of our alpha diversity measures, Chao, Shannon, and inverse Simpson if a multivariable model was used (Table 3). For Chao, similar results were observed for both the univariable and multivariable models. The pre-pregnancy BMI category was strongly associated with the alpha diversity in both univariable and multivariable models for the Chao1 index. For Shannon, the pre-pregnancy BMI was significant in the univariable model, but only the maternal age remained significant in the multivariable model. With inverse Simpson, the pre-pregnancy BMI was associated in the univariable model, and only the maternal age was significant in the multivariable model.

#### 3.5.2. Beta Diversity

The univariable beta diversity results were compared to those of the multivariable models to assess the influence of covariables for both Sorensen and Bray–Curtis PERMANOVA (Table 4). Any variables that demonstrated an association with univariable beta diversity were included in the multivariable analysis for beta diversity. While the overall Sorensen multivariable model was significant (*p* < 0.05), no single factor was significant, but the BMI category contributed the most variance (r^2^ = 0.03), in the multivariable model. The overall Bray–Curtis multivariable model was significant (*p* < 0.01), and college graduation contributed the most variance (r^2^ = 0.04) in the model.

## 4. Discussion

In this study, we establish that pre-pregnancy BMI is associated with various prenatal characteristics and with third-trimester fecal microbial diversity. Women with an obese BMI were significantly more likely to have ever used antibiotics, have ever smoked, be unmarried, not graduated from college, and not own bonds. Women with obesity also had significantly lower alpha diversity, while women with overweight had significantly different bacterial taxa in their microbiomes. The main contributor to the differences in community structure, measured by the beta diversity, was the relative abundance of *Prevotella*, as women who were overweight had a significantly lower percentage. After controlling for any prenatal characteristics that were significantly different within our population, pre-pregnancy BMI remained associated with third-trimester microbial alpha and beta diversity.

Specific prenatal characteristics are associated with the maternal pre-pregnancy BMI, including antibiotic use, smoking status, marital status, education level, and bond ownership status. Antibiotic use has previously been linked to the prevalence of obesity in both human and animal trials [39,40,41]. Our study found that women who were overweight or obese were significantly more likely to use antibiotics before their pregnancy. This coincides with previous research, which has provided evidence that using antibiotics alters the gut microbiome and may decrease the number of bacteria that are known to protect against obesity [42,43]. Antibiotics disrupt the normal function of the gut microbiome, causing dysbiosis, altering nutrient and vitamin intake [44]. Similarly, we found that women with obesity in our study population were significantly more likely to have ever smoked. However, there is conflicting evidence on the associations between BMI and smoking status. Some studies have found that obese adults are significantly more likely to also be smokers [45], while others have found that smoking may be associated with a lower BMI [46]. Women with obesity in our study were more likely to be unmarried; a previous study in the United States of America found that married women were 21% less likely to be obese [47]. More research needs to be conducted to understand the mechanisms behind BMI and its relation to both smoking status and marital status. Furthermore, the overall evidence points to education level having an inverse relationship to BMI [48]. One study found that, for each additional year of education a person receives, their likelihood of obesity decreases between 2 and 9% [47]. However, some findings suggest that ethnicity plays a role in determining how one’s socioeconomic status is related to obesity [49]. It has been shown that the relationship between socioeconomic status and obesity is much more apparent in white populations compared to ethnic minorities [49]. These connections may help explain our results, as the majority of the study population was white (88.1%), and women with obesity in our study were significantly less likely to be a college graduate. Lastly, our results suggest that women with obesity are significantly less likely to be bond owners. One study also found that bond ownership is associated with a normal BMI [50], but there are limited findings in this area of study. Overall, the relationship between obesity and specific prenatal characteristics is complex.

The maternal pre-pregnancy BMI was significantly associated with both the alpha and the beta diversity of the microbiota of pregnant women during their third trimester of pregnancy. While there was no difference in the richness (Chao) of the fecal microbial communities between different BMI groups, women with obesity did have different levels of evenness (inverse Simpson, more emphasis on dominant taxa) and diversity levels (Shannon, accounts for rare taxa). Findings on the associations between the gut microbiota and maternal pre-pregnancy BMI are inconsistent. Some studies have found little to no association between the two [51,52], while others have established clear microbial differences between BMI categories, aligning with our results [14,53,54]. Additionally, associations between the gut microbiota alpha diversity and the pregnancy BMI are mixed. A prior meta-analysis observed that of seven studies that analyzed the Chao1 index of obese versus non-obese individuals, three studies reported lower richness levels while the other four found higher richness or no association [17]. Our results indicate that our study population of women with obesity is one subset that has normal richness compared to other BMI groups. In studies that have previously amplified the V3-V4 region, half (six of twelve) observed a lower Shannon index in obese versus non-obese individuals [17]. The association between low alpha diversity and obesity is most strongly observed in non-Hispanic white populations [55], like our cohort. Other research suggests that this difference in microbial diversity levels may be explained by dietary components, like fiber, meat, and fat intake. Individuals who consume large amounts of high-fat red meat have been shown to have a low alpha diversity and the highest BMI levels [55]. Maternal age is another variable that was significantly associated with evenness (inverse Simpson, abundant taxa) and diversity levels (Shannon, rare taxa) in our alpha diversity analysis. The relationship between maternal age and gut microbial diversity is not well characterized, but some studies suggest that the alpha diversity is higher in the gut microbiomes of young adults [56]. Our alpha diversity results support the idea that increased maternal age is associated with a greater evenness (inverse Simpson) and lower diversity levels (Shannon). Women with obesity in our study also had significantly different microbial community composition (Sorensen index) and structure (Bray–Curtis) compared to normal and overweight women.

When investigating specific bacterial taxa differences, women with overweight were the only maternal pre-pregnancy BMI category that had a difference in the relative abundance of bacteria, specifically *Prevotella.* Previous evidence has linked *Prevotella* abundance to high fiber intake, smoking, as well as lower alpha diversity [57,58]. These results indicate that the microbial differences of the women with obesity in our study may be due to factors such as genetics, lifestyle, or diet. Our analysis also measured the *Firmicutes*-to-*Bacteroidetes* ratio, which has been considered a hallmark indicator of obesity [59]. However, the *Firmicutes*-to-*Bacteroidetes* ratio has come to be considered highly variable and has not been scientifically correlated to any specific metabolic process or mechanism [60]. There was no difference in the *Firmicutes*-to-*Bacteroidetes* ratio among women in the three pre-pregnancy BMI categories tested in our study, which indicates that this measure is not an effective indicator of obesity for our study population.

Smoking status, asthma diagnosis, and marital status were associated with the abundance of several bacterial taxa in our study. A participant’s smoking status and asthma diagnosis was associated with their relative abundance of *Blautia,* with smokers and asthmatics having higher relative abundances. There are discrepancies in the findings regarding *Blautia* levels for individuals with obesity, with evidence of higher levels of *Blautia* in both obese individuals [53,61] and non-obese individuals [62]. *Blautia* is one of the most abundant genera in the gut, regardless of race [63], but it is less prominent in diabetic adults and in those with other diseases [62]. Further evidence suggests that high-protein and low-carbohydrate diets or diets mainly composed of animal products can decrease an individual’s number of *Blautia* species [64,65]. Our data also show that *Lachnospiraceae* was a bacterial taxon that was significantly more abundant in married women in our cohort. Members of the *Lachnospiraceae* family have been associated with both type 2 diabetes and obesity [66]. Though, the married women in our cohort were less likely to have obesity. Further investigation into the health history of the participants is required to assess whether the differences in bacterial taxa can be attributed to their prenatal characteristics or other health factors.

There were some limitations within our study that may have impacted the validity of our findings. One limitation was that the height and weight were self-reported. Self-reported pre-pregnancy height and weight have been found to be accurate in past female populations [67], but, in more recent studies, women with obesity have been found to be slightly more likely to overreport height and underreport weight [68]. Due to a small sample size and a limited number of non-white participants, the generalizability of our findings is limited for larger populations. The composition and functionality of the gut microbiome varies greatly between non-Hispanic whites and racial minority groups [69,70]. The inclusion of more participants from additional racial groups would provide a more representative dataset and allow for better generalizability. Furthermore, as an observational study, we are unable to assess the long-term impacts and changes of the gut microbiome both before and after pregnancy. A longitudinal study with a large sample size, such as the NIH Environmental Child Health Outcomes cohort, would allow us to determine whether our results were heavily influenced by short-term factors, like diet or exercise levels, compared to long-term factors, like BMI or medical history [71,72]. Future studies should also assess whether the microbial composition at the phylum level is associated with the pre-pregnancy BMI.

Additionally, dietary intake, physical activity, and health history data were not included in this analysis of the participants in our cohort. Differences in diet, exercise levels, and medical history have been shown to play a key role in shaping the human gut microbiome and epigenetic profiles [10,51,73,74,75]. Pregnant women with a preference for a “Western” diet, including large amounts of red meat, processed food, and alcohol, have previously been associated with higher pre-pregnancy BMI and excessive gestational weight gain [76,77]. More specifically, maternal nutrition plays a vital role in providing key nutrients, such as folate, methionine, and choline, which are required for one-carbon DNA methylation [13]. A maternal high-fat diet during pregnancy has been shown to lead to gut dysbiosis and increased exposure to pro-inflammatory markers involved in epigenetic aging [10,78]. Recent clinical trials have found that probiotic supplementation in pregnant women can lead to significantly decreased levels of DNA methylation in genes associated with obesity in both mothers and their infants [79]. While our study did not record nutritional trends, future studies should aim to investigate how the maternal gut–brain axis affects infant microbiota colonization and subsequent epigenetic aging.

## 5. Conclusions

This study reports associations between maternal pre-pregnancy BMI and prenatal characteristics as well as the gut microbiome. Prenatal characteristics, such as having ever smoked, marital status, antibiotic use, education level, and bond ownership, were associated with the BMI. Both the alpha and the beta diversity of the pregnancy gut microbiota were significantly different between BMI categories and across various other prenatal characteristics. Specific taxa, including *Prevotella,* were associated with the BMI, while *Blautia* and *Lachnospiraceae* were associated with various other prenatal characteristics. We hypothesize that differences in specific bacterial taxa may be explained by genetic, environmental, dietary, or lifestyle factors, but more health history is required to assess the validity of this hypothesis. Understanding the connections between prenatal characteristics, BMI, and the maternal microbiome, provides insight into how specific bacteria or diversity in the gut microbiome might be used as indicators of health risks or pregnancy outcomes.

## Figures and Tables

**Table 1:** Characteristics of third-trimester pregnant participants by BMI status.

**Pre-Pregnancy ** **Characteristic**	**Normal BMI** **(n = 44)** **n, (%)**	**Overweight BMI** **(n = 17)** **n, (%)**	**Obese BMI** **(n = 23)** **n, (%)**	***p*-Value**
Race				0.371
White	40 (90.9)	16 (94.1)	18 (78.3)	
Black	2 (4.5)	1 (5.9)	4 (17.4)	
Other	2 (4.5)	0 (0)	1 (4.3)	
Antibiotic use *	13 (29.5)	12 (70.6)	16 (69.6)	0.001
Ever smoked cigarettes	2 (4.5)	1 (5.9)	7 (30.4)	0.006
Ever smoked marijuana	3 (7.0)	1 (6.3)	3 (13.0)	0.657
Missing, n (%)	1 (2.3)	1 (5.9)	0 (0)	
Asthma diagnosis	5 (11.4)	5 (29.4)	6 (26.1)	0.165
Allergy diagnosis	4 (9.3)	2 (11.8)	2 (8.7)	0.943
Missing, n (%)	1 (2.3)	0 (0)	0 (0)	
College graduate	34 (77.3)	13 (81.3)	11 (47.8)	0.024
Missing, n (%)	0 (0)	1 (5.9)	0 (0)	
Married	39 (88.6)	12 (70.6)	12 (52.2)	0.004
Owns a car	43 (97.7)	14 (82.4)	19 (82.6)	0.060
Owns a home	33 (76.7)	11 (64.7)	16 (69.6)	0.607
Missing, n (%)	1 (2.3)	0 (0)	0 (0)	
Owns stocks or bonds	24 (54.5)	7 (41.2)	5 (22.7)	0.048
Missing, n (%)	0 (0)	0 (0)	1 (4.3)	
**Pre-Pregnancy** **Characteristic**	**Mean (± SD)**	**Mean (± SD)**	**Mean (± SD)**	***p*** **-Value**
Maternal age	31.5 (5.1)	32.8 (7.0)	30.7 (4.1)	0.293
Missing, n (%)	2 (4.5)	0 (0)	1 (4.3)	
Parity	1.38 (1.7)	1.06 (1.1)	2.05 (1.6)	0.129
Missing, n (%)	2 (4.5)	0 (0)	1 (4.3)	

Note: Statistical tests did not include the “missing” category. * Participants were asked to report any antibiotic/medication usage within the past year.

**Table 2:** Alpha diversity of the fecal microbiota of pregnant participants by pre-pregnancy characteristics.

Pre-Pregnancy Characteristics	Chao Mean (SD)	Shannon Mean (SD)	Inverse Simpson Mean (SD)
Normal BMI	116.7 (30.6)	2.7 (0.4)	9.7 (3.6)
Overweight BMI	110.1 (21.4)	2.7 (0.3)	9.8 (3.5)
Obese BMI	102.0 (19.5)	2.5 (0.4)	7.5 (3.3)
*p*-value	0.156	**0.032**	**0.035**
≤31 years of age	106.4 (21.7)	2.5 (0.4)	7.7 (3.6)
>31 years of age	114.0 (26.3)	2.8 (0.4)	10.4 (3.1)
*p*-value	0.152	**<0.001**	**<0.001**
College graduate	113.4 (27.8)	2.7 (0.3)	9.7 (3.1)
Did not graduate college	105.3 (23.3)	2.5 (0.4)	7.5 (3.8)
*p*-value	0.176	**0.020**	**0.014**
Married	111.5 (25.0)	2.7 (0.3)	9.7 (3.3)
Unmarried	111.0 (32.0)	2.5 (0.4)	7.4 (4.1)
*p*-value	0.635	**0.007**	**0.025**
Owned bonds	111.1 (23.2)	2.8 (0.2)	10.6 (2.6)
Did not own bonds	111.5 (29.5)	2.5 (0.4)	8.0 (3.8)
*p*-value	0.696	**<0.001**	**<0.001**

Note: *p*-values in bold are statistically signifciant at *p* < 0.05.

**Table 3:** Multivariable linear regression modeling results for alpha diversity indexes according to pre-pregnancy characteristics.

	Model (Overall *p*-Value)	Variable	Regression Coefficient	Standard Error	*p*-Value
Chao1 Index	Model 1 (*p* = 0.098)	Intercept	114.098	3.708	<0.0001
		Normal BMI	-	-	-
		Overweight BMI	−3.987	6.907	0.565
		Obese BMI	−11.536	6.324	0.072
	Model 2 (*p* = 0.444)	Intercept	109.330	6.831	<0.0001
		Normal BMI	-	-	-
		Overweight BMI	−5.625	7.693	0.467
		Obese BMI	−14.752	7.705	0.059
		Antibiotic usage	2.084	6.420	0.746
		Smoking status	11.445	9.360	0.225
		College grad	−0.243	7.107	0.973
		Marital status	−1.831	7.914	0.818
		Maternal age	8.477	6.050	0.166
Shannon Index	Model 1 (*p* = 0.076)	Intercept	2.677	0.058	<0.0001
		Normal BMI	-	-	-
		Overweight BMI	0.063	0.108	0.561
		Obese BMI	−0.192	0.099	0.057
	Model 2 (*p* = 0.006)	Intercept	2.596	0.099	<0.0001
		Normal BMI	-	-	-
		Overweight BMI	0.116	0.112	0.305
		Obese BMI	−0.122	0.112	0.279
		Antibiotic usage	−0.085	0.093	0.367
		Smoking status	0.227	0.136	0.100
		College grad	0.021	0.103	0.837
		Marital status	−0.198	0.115	0.089
		Maternal age	0.197	0.088	0.028
Inverse Simpson Index	Model 1 (*p* = 0.047)	Intercept	9.553	0.543	<0.0001
		Normal BMI	-	-	-
		Overweight BMI	0.279	1.012	0.783
		Obese BMI	−2.122	0.543	<0.0001
	Model 2 (*p* = 0.006)	Intercept	8.722	0.935	<0.0001
		Normal BMI	-	-	-
		Overweight BMI	0.440	1.053	0.677
		Obese BMI	−1.651	1.055	0.122
		Antibiotic usage	−0.245	0.879	0.781
		Smoking status	1.133	1.281	0.379
		College grad	−0.116	0.973	0.905
		Marriage status	−1.608	1.083	0.142
		Maternal age	2.274	0.828	0.008

Note: Hyphens indicate the reference group for variables with more than two levels.

**Table 4:** Beta diversity permutational multivariable analysis of variance using distance matrices according to pre-pregnancy characteristics.

Beta Diversity Model (Overall *p*-Value)	Variable	*p*-Value	R^2^	Overall R^2^	F-Statistic
**Sorensen ** (*p* = 0.024)	BMI	0.113	0.031	0.080	1.348
	Antibiotic usage	0.480	0.011		
	Smoking status	0.353	0.013		
	College graduation status	0.082	0.018		
Bray–Curtis (*p* = 0.007)	BMI	0.109	0.034	0.103	1.792
	Antibiotic usage	0.490	0.010		
	Smoking status	0.489	0.010		
	College graduation status	0.003	0.041		

## Data Availability

The data presented in this study are available on request from the corresponding author. The data are not publicly available due to the sample size and consortium requirements.
